# Five Challenges When Managing Mass Casualty or Disaster Situations: A Review Study

**DOI:** 10.3390/ijerph17093068

**Published:** 2020-04-28

**Authors:** Karin Hugelius, Julia Becker, Annsofie Adolfsson

**Affiliations:** 1 School of Health Studies, Örebro University, 701 82 Örebro, Sweden; annsofie.adolfsson@oru.se; 2 Institute for Disaster and Emergency Management, 141 69 Berlin, Germany; juliabeckercp@gmail.com

**Keywords:** mass casualty situation, crises management, disaster management, incident command, contingency planning, disaster response

## Abstract

*Background*: Managing mass casualty or disaster incidents is challenging to any person or organisation. Therefore, this paper identifies and describes common challenges to managing such situations, using case and lessons learned reports. It focuses on sudden onset, man-made or technologically caused mass casualty or disaster situations. *Methods*: A management review was conducted based on a structured search in the PubMed and Web of Science databases. *Results*: The review included 20 case—and lessons learned reports covering natural disasters, man-made events, and accidents across Europe, the United States of Amerika (USA), Asia and the Middle East. Five common challenges were identified: (1) to identify the situation and deal with uncertainty, (2) to balance the mismatch between the contingency plan and the reality, (3) to establish a functional crisis organization, (4) to adapt the medical response to the actual and overall situation and (5) to ensure a resilient response. *Conclusions*: The challenges when managing mass casualty or disaster events involved were mainly related to the ability to manage uncertainty and surprising situations, using structured processes to respond. The ability to change mind set, organization and procedures, both from an organizational- and individual perspective, was essential. Non-medical factors and internal factors influenced the medical management. In order to respond in an effective, timely and resilient way, all these factors should be taken into consideration.

## 1. Introduction

Mass casualty and disaster situations may literally turn life upside down for the people affected; and the number of man-made disasters—such as major transport accidents, terror attacks, fires and other natural disasters—has increased dramatically [1]. Crisis management is a systematic attempt, made by organisational members and external stakeholders, to avert crises or to effectively manage those that do occur [2]. Emergencies, mass casualty situations, catastrophes or disasters are some of the terms for situations that cannot be managed by normal procedures. A mass casualty incident involves a large number of injured people but can generally be managed with the affected organisation’s resources [3]. Disasters, on the other hand, require assistance from external partners. This paper will consider both mass casualty events and disasters without distinction since all such situations challenge healthcare systems and must be managed through specific strategies. Several concepts, which vary considerably, have governed prehospital and hospital management of mass casualty and disaster situations, focusing on command and communication structures [4] or conceptualisation and applications for surge capacity models [5,6]. However, the reality of managing any mass casualty or disaster situation is more complex, and medical responses must be further explored from a crisis management perspective [7,8]. This review will, therefore, move beyond conceptual strategies and focus on experiences from the practical management of mass casualty incidents and disasters. Thus, the paper, from a practical perspective, will identify and describe common challenges for managing mass casualty and disaster situations, using case and lessons learned reports.

## 2. Method

A crisis management review was implemented [9], based on case reports from sudden onset, man-made or natural disasters and mass casualty incidents. First, the authors conducted a structured literature search in the PubMed and Web of Science databases using the following search terms and Boolean tools: ‘disaster OR mass- casualty *’ AND ‘case-report OR lessons learned’ and ‘experiences’. A free text search was also run for: ‘terror attack’, ‘flight crash’, ‘bus accident’, each combined with ‘case report’ or ‘lessons learned’ or ‘experiences’. Table 1 shows the search results (see Table 1).

**Figure 1:**
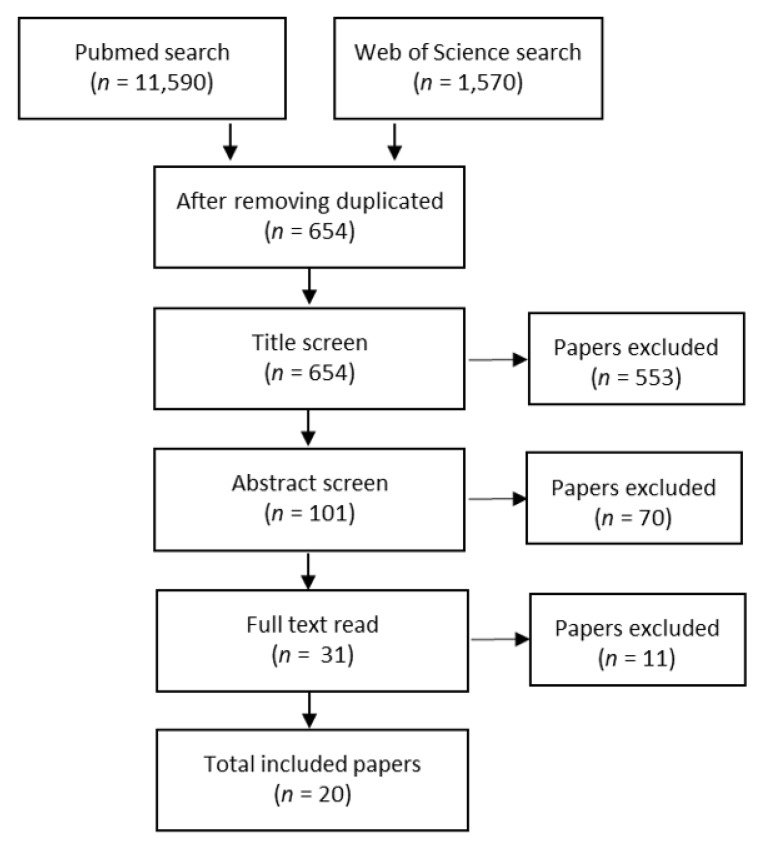
Overview of the process to identify papers for the review.

To be included, a paper was required to describe a mass casualty incident caused by a sudden onset man-made, technical or natural event and report, from a local perspective, how the event was managed. Papers written in English and published between 2009–2019 were included. Papers describing editorial texts, literature reviews or trainings were excluded, as were those describing events from the victims’ perspective or the perspectives of foreign medical teams (see Figure 1). In all, 20 qualifying papers were identified, and their general quality was appraised [9]. If a paper presented a clear aim and described its data/sources and its methodology for data evaluation, the paper was deemed to be of acceptable quality (AQ). If the paper provided information about its data collection procedures, described its analysis process (including statistical methods or methods for synthesising qualitative data) and ethical considerations were addressed, the paper was deemed to be of high quality (HQ) (see Table 2). No paper was excluded from the review due to low quality (LQ).

This analysis process was inspired by the integrative review analysis process [30,31]. All texts describing lessons learned, experiences, challenges or recommendations were extracted, classified according to their content, displayed and sorted by their content into clusters. Thereafter, the content of all clusters was compared, and a pattern of five themes appeared, describing challenges in the practicality of crises management. The findings were conceptualised, and each primary paper was reviewed in its entirety to verify that the new conceptualisation was congruent with the primary sources.

## 3. Results

In all, 20 papers reporting lessons learned or experiences from mass casualty or disaster situations around the world were included in the review (see Table 2). All papers reported from the local management perspective, even if some of the events covered had required international support. Five categories emerged, describing operational challenges when managing mass casualty incidents or disasters. Table 3 provides an overview of how the included papers reported each category as a challenge.

### 3.1. Challenge 1: To Identify the Situation and Deal with Uncertainty

Dealing with uncertainty, unpredictability and unexpected consequences was a significant challenge in many of the described events [12,15,16,22,25,26]. Unconfirmed information, a lack of information and contradictory information cause blurred pictures that made decision-making more difficult than in normal situations. Questions concerning the number of injured or affected people were central in the immediate phase of the mass casualty or disaster events. Combined with obvious difficulties in determining the numbers, this led to significant frustration and made it difficult to assess whether the situation was, in fact, a mass casualty or disaster [12,26]. Sometimes, the contingency plan or disaster mode was not activated until long after the first patients were already in the emergency departments [10,15,24,25].

Unexpected events, and unexpected consequences from those events, especially in the immediate phase, surprised healthcare services, at both operational and strategic levels, and demanded improvisation from management and bedside health professionals [13,19,22,23,26]. Health professionals were overwhelmed by situations that were more complex and stressful than expected, and they felt unprepared and uncertain of what to expect and what to do [17].

### 3.2. Challenge 2: To Balance the Mismatch Between the Disaster Contingency Plan and the Reality

All 20 papers discussed the use of disaster contingency plans. One challenge of specific interest was balancing following the plan or improvising, both individually and organisationally [10,12,14,21]. In some cases, the plan did not cover the right things, or it was not applicable to the situation because the public did not behave as the plan presumed or the event developed in an unexpected direction [10,21,24]. One example of this was that spontaneous evacuation—injured people not awaiting prehospital medical care, but leaving the disaster site on their own or with the help of bystanders to go to the hospital—was not mentioned in any plan but occurred in many of the studied events [10,12,15,25,27]. This, combined with strained prehospital medical care, resulted in victims not being ‘prepared’ (i.e., pre-treated) as expected by the contingency plans. This caused a mismatch between the contingency plans and the need for improvisation in emergency departments [25]. The disaster contingency plans, in general, mainly focused on severely injured patients. However, in many of the studied events, most patients needed basic medical care and/or psychosocial support [10,12,25,26]. The patients arriving at the hospital required, not only surgical care, but also internal medicine care [10,21,28]. An unexpected inflow of patients with chronic diseases, due to lack of electricity or medical instruments (e.g., home care ventilators or medications) also occurred [23]. Addressing such complex medical needs was not covered in the contingency plans [10,12,26]. Lack of equipment was not reported in any of the included events, but, in some, the equipment specifically stored for disaster use did not match the actual needs [23,26]. For example, stretchers and blankets were the most used and requested equipment after the terrorist attack in Norway, but the stored equipment was mainly surgical equipment [26].

### 3.3. Challenge 3: To Establish a Functional Crisis Organisation

A functional crisis organisational structure was the foundation for managing these sampled mass casualty or disaster incidents. Essential to this management, both prehospital and in hospital, were: designing a clear crisis organisational and command structure, having a strong and skilled incident commander and ensuring that all persons and stakeholders involved understood the structure (e.g., chain of command) and acted as directed [12,18,24,27,28]. Also, to function, the crisis organisation needed to be simple and robust [15,19]. On the other hand, lack of knowledge and experience about how to work in a crisis organisation, using command and control structures or other specific methods (e.g., international humanitarian cluster coordination or staff procedures), reduced the effectiveness of response to and management of situations [27].

A very common problem was that communication failed, causing significant challenges for functional crisis response. Examples of communication failures were technical, such as power cuts or overloaded mobile networks [10,16,19,26,27,28] lack of proper information management, reduced methods of confirming information, rumors [23], as well as language barriers and various interpretations of terms (e.g., ‘severely injured’) [26,27]. To correctly analyse the consequences of an event and the effectiveness of a response, feedback and information from the people on the ground and the people affected, was necessary, but could not be obtained because means of communication were lacking [14,23,28]. In this way, communication problems affected, not only medical responses, but also strategic decisions.

No report considered a lack of health professionals to be a major problem. However, an overabundance of health professionals willing to serve, but not needed for the moment, turned into a problem that demanded valuable resources from the crisis organization [10,15,23].

### 3.4. Challenge 4: To Adapt the Medical Response to the Actual and Overall Situation 

The sampled mass casualty or disaster events demanded that health professionals adapt to unusual contexts and work in unusual ways. For example, they had to act and provide medical care under threat or in hostile environments [13,26] or change the way procedures were carried out (e.g., because of darkness or cold) when working outside [20]. Also, some interventions that were usually conducted by one specialist had to be conducted by others, such as nurses suturing or prescribing analgesics or antibiotics [16]. In some cases, patients who would, under normal circumstances, have been treated in non-surgically or with more sophisticated surgical techniques were treated with faster, simpler surgical methods because opportunities to closely monitor patients were limited [22,28]. Not only organizational structure, the flow of patients or management was adjusted, but also the protocols on how to examine and treat patients [10,13,26,28]. Adjusting numbers of staff members or the medical specialities each member performed was common. For example, trauma teams, which normally contained 13 medical professionals, consisted of one medical doctor and one nurse [25]. Orthopaedic surgeons dealt with infectious diseases [28] and gynaecologists with general trauma patients [26]. Such adjustments required all health professionals to have sufficient general medical knowledge and the personal courage to accept the necessary adaptations.

A health professional’s role could also change from providing direct, hands-on treatment to supervising others, such as unexperienced colleagues, volunteers, bystanders or relatives [12,15]. Additionally, the usual digital medical record systems were unavailable – either because they could not cope with the sudden patient influx or because healthcare was being administered in unusual locations. Therefore, documentation was reverted to pen and paper procedures [22].

Several logistical problems greatly influenced on both medical response and health outcomes from the sampled events [10,14,22,23,26,27,28]. These ranged from geographical distances, inaccessible terrain and destroyed infrastructure (e.g., roads or telecommunications) to traffic jams caused by evacuations or weather conditions. One question, highly influenced by logistical, infrastructural factors and local geographical knowledge was patient distribution [15,22,24,26]. Sometimes, the disaster contingency plan stated that the injured should be dispersed from the prehospital scene to several hospitals. However, in such instances, due to unexpected factors—such as reduced resources for transports, spontaneous evacuation and security concerns—all patients had to be taken to one hospital [15,22]. This strategy was advantageous because it demanded less transport capacity and, therefore, gave providers the opportunity to provide advanced prehospital medical care; it also enabled the reunion of families and friends, which eased the burden for medical staff [21,22]. During other events, patients with the same types of injures were transferred to the same hospitals, followed by suitable specialists. This strategy reduced the need for referrals at a later stage [29].

When patients were discharged from hospitals to free beds for victims of a mass casualty or disaster event, the burden on community-based healthcare increased. Combined with infrastructure problems, this created a significant, unexpected burden on the overall medical system [14,23]. To cope with this, proactive health education, with self-care advice and community engagement, was offered, and the public complied well with the advices provided [27].

### 3.5. Challenge 5: To Ensure a Resilient Response

Most often, the mass casualty or disaster events affected the medical organisation for longer than expected or planned [13,17,28]. An increased need for general healthcare, rehabilitation and psychosocial support lasted for several months or years [10,12,26].

However, the victims were not alone in being affected by the events. Health professionals were also affected, sometimes personally (e.g., losing family members) [13,16,17,23]. Even if they were not personally affected, the psychological stress they endured reduced their capacity to function, both in the immediate aftermath and for some time to come [12,18,23,24]. Professional psychosocial assistance was sometimes offered to promote the health professional’s recovery [12,15,17]. This long-term burden on the healthcare system, combined with the reduced capacity of the staff, necessitated staff planning. At the same time, the overabundance of health professionals spontaneously reporting to serve in the immediate aftermath caused chaos and limited the possibilities for long term planning [23]. Another point requiring staff planning was the clean-up, resupply of equipment and waste disposal when the immediate phase had passed, and normal operations were supposed to resume [15].

Several strategies were used to streamline overall response capacities. The sampled events indicated that requests for national or international assistance should be made early if they are to be useful [13,20]. Such assistance was used to reinforce response organisation, to add valued expertise or experience, to enable rest and recovery for the professionals deployed in the very first stage, or (with medical institutions) to refer patients away from the disaster area and, thereby, ease the burden on the affected area’s healthcare system [13,20,23]. Voluntary resources were contributed after many of the sampled events, such as people providing food or offering general social support to the victims. Their efforts added value to the disaster response organisation, if the organisation was prepared to manage those efforts. However, if unprepared, these efforts added further problems to the already stretched organization [10,11,14,23,29].

Another area challenging the resilience of the response was the hospitals and associated buildings had been constructed. Despite contingency plans and special preparations, such as ‘earthquake safe buildings’, severe shaking, in some cases, caused long power cuts, damaged buildings and made it necessary to evacuate, for example, the emergency and ambulance dispatch centres, as well as parts of the hospitals and management centrals [10,11,23].

## 4. Discussion

This review of 20 case studies or lessons learned reports from sudden onset mass casualty and disaster situations has shown that managing such situations involves several challenges. These challenges are related to the ability to manage uncertainty, a lack of conformity between the contingency plans and the real situation, ineffective crises management organisations and ineffective information management, as well as the adaptation of medical and non-medical factors to ensure resilient crisis response.

Being prepared for the unknown is an important part of disaster management [32], and this review confirms that unplanned and surprising situations occurred in all sampled events. The ‘black swan’ metaphor [33]—things that have never happened before or seem unrealistic actually do occur—is, therefore, an adequate reference for the mental preparation and training necessary to adequately manage disasters. The gap between the disaster plans and the real events found in this review were obvious and deserve further attention. However, successful disaster management is not based on plans, but on activity [34]. Also, this review showed that not only medical perspectives and skills are needed in a successful disaster response, but also non-medical perspectives and factors strongly influenced the decision making and response. Therefore, a cross agency planning and collaboration in the response is needed. One critical moment in managing mass casualty or disaster events is the establishment of a functional crisis organisation, changing from everyday procedures to disaster mode procedures. The review showed that most often, there was no, or a very short, time span between the first signals of a potential disaster situation and the resultant influx of patients. Dealing with uncertainty and the lack of confirmed information, such as numbers of the injured, is crucial. Most disasters are unpredictable, uncertain and dynamic [35]. Being able to use an intuitive sense or ‘gut feeling’, rather than exact information, is an important personal skill among incident commanders [4,36]. When analysing the included papers, it was evident that the stated numbers of dead, injured and affected people differed from paper to paper, even if they described the same event. Also, terms like ‘severely injured’ or ‘affected’ were used but not defined. This is an example of the lack of conformity that exists in reporting mass casualty or disaster experiences, both in life and in science, which can confuse and challenge situation management. Communication problems were reported in all included papers. However, the problems were of various kinds, ranging from technical flaws to information management issues. Robust backup systems for transferring information, as well as information management procedures and skills are necessary to create a functional and adequate crises response.

Surge capacity is a term often used in disaster response to illustrate the ability to evaluate and care for a markedly increased volume of patients. One part of that is to change patient flows. However, the review also illustrates that it became necessary, in the sampled events, to use ‘old fashioned’ or unsophisticated medical interventions or to trust clinical decisions without laboratory tests, monitoring or x-rays. This flexibility and adaption of medical procedures demands that all health professionals have general medical competence and good clinical skills- and are trusted to do so. Today, many health professionals are not accustomed to using primitive methods or working without advanced technology [37]. Also, the growing trend to sub-specialise in a specific field of medicine may increase the general vulnerability of medical services in disasters. Altered methods of performing medical procedures, compared to everyday situations, may also explain some of the reported mismatch between stored equipment and the kind of equipment needed in the real mass casualty or disaster situation. Further studies on health consequences from different types of mass casualty and disaster events, as well as the adapted medical procedures implemented in these cases, are necessary to increase the knowledge of what medical disaster supplies are actually needed from both a short- and long-term perspective.

It is well known that the health effects of mass casualty and disaster events include physical injures, potential severances of chronic or internal medicine conditions and psychosocial problems (e.g., stress reactions, post-traumatic stress syndrome (PTSD), depression, disturbance in social relations and economic consequences) [38,39]. Many of the included papers focused on physical injures and surgical management, but victims’ psychosocial needs were also addressed as an important issue that consumed significant efforts in healthcare services. Physical and psychosocial medical care should be integrated into disaster responses to make those responses effective [38,39,40]. The present review did not specifically analyse psychosocial support, but, given that most papers highlighted this perspective, organising and providing psychosocial support after mass casualty and disasters deserves further attention.

From the perspective of a resilient healthcare system, the wellbeing of health professionals is essential. Since many of the studied events reduced health professionals’ capacities due to stress, their wellbeing should be considered and integrated in planning, the emergency phase and the post-emergency aftermath [41]. However, there is no consensus on how to prepare and support professionals involved in potentially traumatic events, and single sessions have been comparatively ineffective at promoting recovery [42,43]. Despite mass casualty situations and disasters having challenged medical services from the beginning, there is still no general agreement about what disaster medicine competence actually is or how health professionals should prepare for disaster situations [44]. However, incident commanders should communicate clearly, have the professional and personal capabilities to build trust among their staff members and be trained and prepared to take full responsibility for the situation, their staff and themselves [4,45].

All disasters and crises are unique. Thus, generalising from one situation to another may be difficult, but this does not discredit the need for lessons learned. Lessons learned are often repeated, time after time, if not cared for beforehand and implemented in the formal organisation and training [46,47]. Sharing personal and professional experiences can also add value to personal mental preparedness and possibly contribute to resilience, both individually and organisationally.

### Limitations

The base of evidence in disaster medicine and crisis management is generally low, and many best practice recommendations are founded on lessons learned and shared experiences [47,48]. However, a method for reporting and evaluating crisis management is not yet standardised. Therefore, the learning process and evidence development must rely on a comprehensive synthesis from experiences rather than traditional, randomised studies [9,49]. As part of a review methodology, several analysis methods may be employed [9]. In this study, the analysis process was inspired by the integrative review methodology—incorporating both quantitative and qualitative data [30]. However, since the review did not rely solely on original research, no standardised quality appraisal of the included studies was made. Building synthesised knowledge from case studies and other types of reports reduce the possibility and value of such an assessment, and, therefore, a comprehensive appraisal of quality is recommended [9]. As in every review, there may be other papers this study did not identify. Additionally, papers were excluded if they did not cover sudden onset, man-made or natural disaster events from a practical medical management perspective. For example, papers covering the Ebola outbreak in Western Africa in 2014 were not included. The reason for this was that a panademic most often is a slowly developing event, compared to explosions or air plane accidents. However, there is no reason to doubt that the majority of the challenges presented in this study would not be valid for panademics also. A synthesis of lessons learned from such slow onset events, for example the Covid 19 panademic and the Ebola outbreak would add further value to current knowledge about the medical management of disasters. It should also be kept in mind that this review covers only 20 out of thousands of mass casualty or disaster events that have occurred over the past ten years. Therefore, the results must be interpreted with some caution, and they do not rule out other challenges that might be present during the management of a mass casualty or disaster situation. In order to further explore successful strategies to respond to mass casualty- and disaster situations, these challenges may provide a basis for further studies.

## 5. Conclusions

The challenges when managing mass casualty or disaster events involved were mainly related to the ability to manage uncertainty and surprising situations and using structured processes to respond in a resilient way. The ability to change both mind set, organization and medical procedures as well as decision-making processes and command structure, both from an organizational- and individual perspective, was essential. Both medical factors, non-medical factors and internal factors such as stress among staff and commanders did influence the medical management and decision making process. In order to respond in an effective, timely and resilient way, all these factors should be taken into consideration in both the planning and operative phase of a medical mass casualty or disaster event. In particular, the short timeframe from event to need for response, the long lasting need for response, the influence of non-medical elements and planning for step down procedures and recovery should be paid specific attention to in order to ensure an effective crises management.

## Figures and Tables

**Table 1:** Overview of literature search.

Database	Search Terms	Number of Records
PubMed database 11 October 2019 Language: English Publication dates: 2009–2019	S1: (disaster OR mass-casualty incident)	31,844
	S2: S1 AND (case-report OR lessons learned)	1118
	S4: (terrorist attack *) AND (experience *) OR (case-report OR lessons learned OR experience)	19
	S5: (flight crash) AND (experience *) OR (case-report OR lessons learned)	9
	S6: (bus accident) AND (experience *) OR (case-report OR lessons learned)	13
	In total	1159
Web of Science 11 October 2019 Language: English Source: Article Publication dates: 2009–2019	S1: (disaster OR mass-casualty incident)	35,271
	S2: S1 AND (case-report OR lessons learned)	1500
	S4: (terrorist attack *) AND (experience *) OR (case-report OR lessons learned OR experience)	29
	S5: (flight crash) AND (experience *) OR (case-report OR lessons learned)	23
	S6: (bus accident) AND (experience *) OR (case-report OR lessons learned)	18
	In total	1570
	After reducing duplicates	654

Note: * was used for truncation.

**Table 2:** Overview of included papers and quality rating.

Authors *	Year of Publication	Type of Event	Number of Affected (Dead/Injured/Not Injured) **	Country	Type of Paper ***	Quality Appraisal ****
Ardagh et al. [10]	2012	Earthquake	182/6659/NA	New Zealand	Review	HQ
Campion et al. [11]	[11] 2016	Airplane crash	3/192/112	USA	Original research	AQ
Carli et al. [12]	2018	Explosion and shooting	96/115/NA	France	Case report	AQ
Carli et al. [13]	2017	Explosion, shooting and vehicle-ramming attack	137/413/NA and 87/458/NA	France	Viewpoint	AQ
Chandler [14]	2016	Storm	130/NA/NA	USA	Original research	HQ
El Sayed et al. [15]	2018	Explosion	3/32/NA	Lebanon	Concept paper	AQ
Hugelius et al. [16]	2017	Storm	8000/45,000/NA	The Philippines	Original paper	AQ
Johal et al. [17]	2016	Earthquake	187/6000/NA	New Zealand	Original Paper	
Safi Keykaleh [18]	2019	Bus accident	11/35/NA	Iran	Case report	AQ
Lesaffre et al. [19]	2017	Explosion and shooting	130/495/NA	France	Commentary	AQ
Lyon & Sanders [20]	2012	Bus accident	28/24/0	Switzerland	Commentary	AQ
Martin et al. [21]	2017	Shooting	7/3/110	USA	Report from the field	AQ
Massalou et al. [22]	2019	Vehicle-ramming attack	86/458/NA	France	Original paper	HQ
Nakagawa et al. [23]	2013	Earthquake and tsunami	9512/3792/NA	Japan	Lessons learned	AQ
Sabah et al. [24]	2018	Explosion	27/227/NA	Saudi Arabia	Original research	AQ
Solla et al. [25]	2018	Vehicle-ramming attack	86/500/NA	France	Commentary	AQ
Sollid et al. [26]	2012	Explosion and shooting	77/NA/NA	Norway	Original research	HQ
Subedi et al [27]	2018	Earthquake	8790/22,300/NA	Nepal	Report from the field	AQ
Zhang et al. [28]	2011	Earthquake	69,000/NA/NA	China	Original research	HQ
Zhang et al. [29]	2018	Explosion	173/798/4000	China	Report from the field	AQ

Note: NA means Not Applicable or reported *—As numbered in the reference list.; **—As reported in the paper.; ***—As classified in the journal.; ****—The grades ‘high quality’ (HQ), ‘acceptable quality’ (AQ) or ‘low quality’ (LQ) were used.

**Table 3:** Overview of the representation of each theme in the included papers.

Author (year) *	Challenge 1: To Identify the Situation and Deal with Uncertainty	Challenge 2: To Balance the Mismatch Between the Contingency Plan and the Reality	Challenge 3: To Establish a Functional Crisis Organisation	Challenge 4: To Adapt the Medical Response to the Actual and Overall Situation	Challenge 5: To Ensure a Resilient Response
Ardagh et al. (2012) [10]	X	X	X	X	X
Campion et al. (2016) [11]					X
Carli et al. (2018) [12]	X	X	X	X	X
Carli et al. (2017) [13]	X			X	X
Chandler et al. (2016) [14]		X	X	X	X
El Sayed et al. (2018) [15]	X	X	X	X	X
Hugelius et al. (2017) [16]	X		X	X	X
Johal et al. (2016) [17]	X				X
Safi Keykaleh (2019) [18]			X		X
Lesaffre et al. (2017) [19]	X		X		
Lyon & Sanders (2012) [20]				X	X
Martin et al. (2017) [21]		X		X	
Massalou et al. (2019) [22]	X			X	
Nakagawa et al. (2013) [23]	X	X	X	X	X
Sabah (2018) [24]	X	X	X	X	X
Solla et al. (2018) [25]	X	X		X	
Sollid et al. (2012) [26]	X	X	X	X	X
Subedi et al. (2018) [27]		X	X	X	
Zhang et al. (2011) [28]		X	X	X	X
Zhang et al. (2018) [29]				X	X

Note: *—The number refers to the reference list number.
